# Posterior Capsule Opacification: A Review of Experimental Studies

**DOI:** 10.3390/jcm10132847

**Published:** 2021-06-27

**Authors:** Joanna Konopińska, Maryla Młynarczyk, Diana Anna Dmuchowska, Iwona Obuchowska

**Affiliations:** Department of Ophthalmology, Medical University of Bialystok, 15-089 Białystok, Poland; mromaniuk2121@gmail.com (M.M.); diana.dmuchowska@umb.edu.pl (D.A.D.); iwonaobu@wp.pl (I.O.)

**Keywords:** posterior capsule opacification, experimental studies, cell cultures, tissue cultures, animal model of PCO

## Abstract

Posterior capsule opacification (PCO) is the most common complication of cataract surgery. It causes a gradual deterioration of visual acuity, which would otherwise improve after a successful procedure. Despite recent advances in ophthalmology, this complication has not been eradicated, and the incidence of PCO can be as high as 10%. This article reviews the literature concerning the pathomechanism of PCO and examines the biochemical pathways involved in its formation and methods to prevent this complication. We also review the reported tests performed in cell cultures under laboratory conditions and in experimental animal models and in ex vivo human lens capsules. Finally, we describe research involving human eyes in the clinical setting and pharmacological methods that may reduce the frequency of PCO. Due to the multifactorial etiology of PCO, in vitro studies make it possible to assess the factors contributing to its complications and search for new therapeutic targets. Not all pathways involved in cell proliferation, migration, and contraction of the lens capsule are reproducible in laboratory conditions; moreover, PCO in humans and laboratory animals may be additionally stimulated by various degrees of postoperative reactions depending on the course of surgery. Therefore, further studies are necessary.

## 1. Background

Cataract is the most common cause of blindness worldwide, and surgical removal is the most common method used to treat it [1]. Cataract surgery has improved in recent years, but postoperative complications still occur with the most common complication being posterior capsular opacification (PCO), which has a multifactorial etiology [2]. PCO not only causes quantitative visual disturbances but also reduces the quality of vision, leading to reduction in contrast sensitivity, halo effect, and lack of binocular vision [3]. Despite improvements in the techniques used in cataract surgery and lens design, statistically, PCO was reported to occur after surgery in approximately 11.8% of cases within the first year, in almost 20.7% by 3 years, and in 28.4% by 5 years [4]. The overall occurrence is as high as 50% in adults and 100% in children from 2 months to 5 years after surgery [5]. In addition to impairing the patient’s quality of life, PCO impedes fundus and specialist ophthalmic examinations, such as optical coherence tomography.

Postoperative capsular opacification is mainly caused by migration and proliferation of residual lens epithelial cells (LECs) after cataract surgery and their differentiation into fibroblastic and lens fiber-like cells. It occurs more frequently and is more severe in young patients than in old, as they exhibit increased numbers of LECs and greater mitotic activity. Therefore, along with the development of improved operating techniques, research is underway to develop an effective and safe method for preventing PCO.

The aim of this research was to systematize the current knowledge concerning the prevention of PCO and to present scientific hypotheses that may contribute to reducing the frequency of this complication. 

### 1.1. Inclusion and Exclusion Criteria

PubMed was searched for suitable publications using the following keywords: “cataract,” “phacoemulsification,” and “posterior capsule opacification.”

The abstracts of the retrieved articles were assessed for relevance, and references were screened to identify those that needed to be manually searched. Publications that were available only as abstracts or conference posters were excluded. In this way, 400 full-text original articles, reviews, randomized clinical trials, and retrospective and cohort studies were included in the study. We included in vitro research, research on laboratory animals, and human clinical trials. None of the relevant articles were excluded based on language. Full-text translation was performed if necessary. Since the journal guidelines restrict the number of references to 100, we chose the studies that we deemed most relevant.

### 1.2. Risk of Bias Assessment

The methodological quality of the included studies was evaluated independently by two authors (I.O. and J.K.). 

### 1.3. Data Extraction

The studies’ demographic details, participant characteristics, interventions, outcomes, and limitations were independently extracted by two authors (I.O., J.K.). When disagreements occurred, they were resolved through discussion.

### 1.4. Risk Factors

The development of PCO is influenced by patient-related factors, factors related to surgical technique, and the type of intraocular lens (IOL) implanted. Patient-related factors include young age, history of uveitis, glaucoma [6], arterial hypertension, metabolic disease, type of cataract (subcapsular), and injury [7,8,9]. These factors are known to increase the risk of PCO in the immediate postoperative period [9], but the mechanism of action has not been completely clarified. Some authors believe that diabetes may play a role in the promotion of PCO [10] while others have suggested that diabetes is a preventive factor [11]. Moreover, the data on PCO are inconsistent regarding sex distribution. The only IOL-related factors proved to be associated with PCO are a rounded edge and the phacoemulsification technique [12]. The geometry of the optic edge of the IOL is also important, as well as the shape and angulation of the haptics. PCO is much less likely to occur with IOLs that have a sharp posterior optic edge (360°). Mian et al., found that the frequency of posterior capsulotomies performed in patients with three-piece lenses was significantly lower than that in those with one-piece lenses [13]. Furthermore, thorough hydrodissection and aspiration of lens masses and polishing the posterior capsule reduce the occurrence of PCO, but increase the procedure time, which may increase the risk of surgical trauma. Incomplete LEC removal may lead to further proliferation of LECs within traumatized tissues, which exacerbates pro-inflammatory processes [14].

### 1.5. Treatment

The most common procedure performed to improve visual acuity that provides insight into the fundus of an eye with PCO is laser capsulotomy [15]. This procedure entails making an opening in the posterior capsule on the visual axis. Laser capsulotomy is accepted as a standard and effective treatment for posterior capsule opacification. It is an effective, quick and relatively easy outpatient procedure, but it can produce some complications. Recent studies have not confirmed its association with retinal detachment, however it may be associated with complications, which range from mild short-term increase in intraocular pressure to visual impairment due to cystoid macular edema, or macular holes [16,17]. Trinavarat et al., noticed that laser capsulotomy may lead to IOL damage, subluxation, dislocation, and secondary glaucoma [18]. Moreover, laser capsulotomy is not compatible with the new generation of accommodative lenses, which require the presence of a lens capsule to function [19]. Laser treatment also places a significant financial burden on healthcare systems and is not widely available in developing countries. Therefore, the second course of action is to search for methods to prevent secondary cataracts. Until now, numerous in vitro and experimental animal studies have been conducted, but many have reported undesirable effects on the corneal endothelium [20,21]. Various trials of antiproliferative therapy have been performed in cells cultured in vitro and lens capsule models or in vivo (in rabbits, monkeys, and humans) [22,23,24,25]. Active agents are delivered to LECs via various carriers (including ophthalmic viscoelastic devices, drug delivery systems [DDSs], or slow-release implants) or can be delivered directly to the cell culture medium during in vitro experiments. Various techniques have also been developed for intraoperative application of pharmacological agents to lens cells, including adding them to infusion fluids, using devices that confine them in the capsular space, or coating the IOLs with the test substance [26].

### 1.6. Pathophysiology

The breakdown of the blood-aqueous humor barrier during cataract surgery causes an immune response. Thereafter, epithelial-mesenchymal transition (EMT) occurs in cells in the lens capsule. The main cause of PCO after cataract surgery is migration and proliferation of LECs. The average number of LECs is approximately 4000–5000/mm^2^, depending on patient age, with a significant decrease in density in those aged 80 years or older. LECs consist of a single layer of cuboidal-cylindrical cells attached to the posterior surface of the anterior lens capsule. A foreign body, such as an IOL, induces an inflammatory reaction, which includes multinuclear leukocytes, giant cells, and fibroblasts, in the anterior chamber in the immediate postoperative period [27,28]. It is worth noting that in animal studies (e.g., in mice from Melinda Duncan’s lab) removal of the lens fiber core also induces the generation of fibrotic cells in the absence of insertion of an IOL. These cells produce cytokines, including transforming growth factor beta (TGF-β), interleukin-1, interleukin-6, basic fibroblast growth factor (bFGF), and tumor necrosis factor alpha (TNF-α), which activate transformation of LECs, proliferation, metaplasia around the equator of the anterior capsule, and migration toward the posterior capsule, leading to thickening and hypertrophy. This process is characterized by fibrosis and contraction reactions due to the activity of actin filaments [29]. Moreover, immunohistochemical studies have identified extracellular matrix (ECM), fibronectin, and collagen molecules on the surface of the IOL [30,31]. Collagen deposition on the IOL and capsule can cause clouding and edema in the posterior lens capsule [31]. 

The extent to which the IOL becomes coated by LECs depends on the material used in the IOL; its surface [31], optic shape, and diameter; mechanical properties of the haptics [30]; and implantation methods used [32]. The two types of PCOs are caused by two different types of epithelial cells. The first type of PCO involves anterior epithelial cells located in the central zone of the anterior capsule, which consist of LECs that are relatively inactive mitotically. When stimulated, these cells are converted to myofibroblasts, causing fibrous metaplasia. Cells may also migrate toward the posterior capsule, where they proliferate and undergo hypertrophy and hyperplasia on the capsule surface, causing opacification [33]. The second type of PCO occurs when pluripotent cells gather around the equator of the capsule. When activated by interleukins, these cells migrate posteriorly. They do not undergo fibrosis but instead form large, balloon-shaped, or clustered Wedl or bladder cells. They undergo constant mitosis, proliferation, hypertrophy, and hyperplasia, creating clouding [34]. They can also participate in the formation of the fibrous form of PCO through the process of fibrous metaplasia, appearing as fibrous membranes. Metaplasia and inflammatory cells of the iris and ciliary body as well as fibrinous exudate also stimulate development of PCO [35]. Furthermore, the residual cortical fibers may regenerate, creating pathological clouding known as Soemmering’s ring and Elschnig’s pearls and is much more common than fibrotic PCO and is the main cause of a decrease in visual function after cataract surgery [31,32].

### 1.7. Cell and Membrane Mediators 

In vitro research uses cells derived directly from human or animal lenses. These cells perfectly reflect the processes occurring during formation of PCO because of the original phenotype of the epithelium and their greater sensitivity to external factors. The proliferation and differentiation capacity of cultured LECs can be assessed using microscopy [26]. Alternatively, cells can be obtained by culture of a cell line; these cells are more stable and can provide information about cellular processes but do not completely reflect all processes occurring under actual biological conditions.

#### 1.7.1. Inhibition of TGF-β

Given that TGF-β plays a key role in the formation of PCO, research efforts have focused on inhibiting its action. Sun et al. [36] performed a study in which they grew human lens epithelial (HLEB3) cells in vitro and stimulated them with TGF-β2. The cells then transformed into fibroblast-like cells and the bonds between them loosened, causing them to migrate (i.e., undergo EMT). Application of a SNAIL small interfering RNA polyclonal antibody released from a coated IOL inhibited the EMT process, mainly via inhibition of migration and adhesion of LECs.

Collison et al. [37] investigated the effect of an anti-TGF-β monoclonal antibody (CAT-152) on progression of PCO. In their experiment, ex vivo human lens capsules were incubated with various concentrations of TGF-β with or without CAT-152. Incubation of the lens capsule with TGF-β resulted in an increase in migration and differentiation of LECs, which, in turn, resulted in shrinkage of the capsule and formation of PCO. Addition of CAT-152 inhibited this process. 

Yang et al. [38] evaluated the ability of pirfenidone to inhibit migration and differentiation of human LECs in vitro and concluded that pirfenidone inhibits TGF-β2-induced cell proliferation and migration, most likely by downregulating the TGF-β receptor (and Smad2, Smad3, and Smad4) in human LECs.

Lovastatin also has the potential to inhibit TGF-β [39]. The mechanism of action of lovastatin involves blocking RhoGTPase, which stimulates the TGF-β-dependent conversion of LECs. In a study performed using a porcine model, LECs were pre-treated with lovastatin and then incubated with TGF-β for 24 h. Unlike the control group, the lovastatin group showed no increase in mRNA expression and protein production of LEC’s, or increased collagen contractility. These findings suggest that lovastatin could be considered for the prevention of PCO [39]. 

Kubo et al. described the roles of TGF and fibroblast growth factor (FGF)-2, their relationship to tropomyosin in regulation of EMT in lens capsule cells, and the development of PCO [40]. They identified two types of TGF-β-dependent differentiation of LECs, with or without FGF-2, based on research performed in mice and rats. The first type led to differentiation of cells in the lens capsule toward tropomyosin-positive myofibroblasts showing epithelial-to-myofibroblast transition (EMyoT) morphology without the presence of FGF-2. The second type differentiated into fibroblastic tropomyosin-negative cells induced by FGF-2 with simultaneous administration of TGF-β2. Increased expression of tropomyosin may be associated with the progression of EMyoT in mice, PCO in rats, and healing of LECs in mice. Tropomyosin is an important marker of PCO and constitutes a therapeutic target in the wound healing process and in neoplastic invasion. Further investigation of the mechanism by which tropomyosin is regulated will provide valuable insights into its inhibitors and introduce new methods of treatment and prevention of EMT-dependent diseases.

Hypoxia-inducible factor (HIF)-1α is degraded after cataract surgery due to an increase in pO_2_. TGF-β2, in turn, increases the activity of HIF-1α in cells after cataract surgery. Nahomi and Nagaraj evaluated the role of HIF-1α in EMT mediated by TGF-β2 in cultured FHL124 human LECs [41]. TGF-β2 was shown to increase the concentration of HIF-1α during EMT in FHL124 cells, while an attempt to increase HIF-1α expression using a prolyl hydroxylase inhibitor did not induce EMT in these cells. Moreover, KC7F2, an HIF-1α protein translation inhibitor, inhibited TGF-β2-mediated EMT in FHL124 cells. These findings indicate that HIF-1α has a significant role in TGF-β2-mediated EMT of LECs, which may be important in prevention of PCO.

Taiyab et al., evaluated the interaction between β-catenin (involved in non-canonical signal transduction) and Smad3 (required in canonical signaling) in TGF-β-induced EMT regulation using an ex vivo rat lens model [42]. Lens cells were tested to show the interaction between the three TGF-β-dependent EMT pathways using alpha-smooth muscle actin (α-SMA). In their study, inhibition of the Smad3 pathway protected against translocation of β-catenin into the nucleus and loss of E-cadherin from cell–cell contacts as well as blocked TGF-β-dependent EMT in lens cells. Inhibition of β-catenin and Smad3 reduced the amount of TGF-β receptor mRNA present. The results of that study indicated a close relationship between interaction in the β-catenin/Smad3 line and regulation of the EMT process in the lens and could lead to development of an agent for the prevention and treatment of PCO.

#### 1.7.2. Disruption of Cell Adhesion

Mibefradil is a T-type antagonist of the calcium channels present in cell membranes. As a non-selective calcium channel blocker, it causes depolarization of cell membranes. Moreover, it reduces the expression of integrins, resulting in inhibition of proliferation and induction of apoptosis. Its use in the prevention of PCO is based on the hypothesis that it inhibits the pathways mediating cell adhesion. The effect of mibefradil dihydrochloride was tested in primary human LECs obtained after cataract surgery and in a human lens cell line (HLE-B3) [43]. During the surgical procedure, fragments of the lens present on the anterior capsule were collected and cultured for 1–2 weeks. They were subsequently separated from the capsule and transferred to a growth medium. Next, the cells were treated with the drug for 24 h. The earliest signs of mibefradil-induced apoptosis of human LECs were observed after 4 h of incubation. These signs were accompanied by a significant reduction in cell size and initiation of apoptosis by phosphatidylserine switch in the plasma membrane. DNA fragmentation in the cell nucleus and fragmentation of cytoskeletal actin were observed. Apoptosis is correlated with inhibition of integrin expression, decreased proliferation, and depolarization of cell membranes. This research suggests that depolarization of the human LEC membrane and inhibition of integrin expression leads to a decrease in cell adhesion and apoptosis, which may inhibit development of PCO. Inhibition of integrins prevents LECs from adhering to the lens capsule and leading to PCO.

Sureshkumar et al. [19] tested the ability of drugs that rapidly inhibit the actin cytoskeleton network to prevent PCO in the human lens capsule. Two agents were used, namely, H-7, a broad-spectrum serine-threonine kinase inhibitor, and latrunculin (LAT)-B, a macrolide isolated from the sea sponge *Latrunculia (Negombata) magnifica*; both agents are less toxic than ethylenediaminetetraacetic acid (EDTA) and the antimetabolites that act in a similar fashion [44]. These substances differ slightly in their mechanism of action in that H-7 reduces focal cell adhesion, damages cell membranes, and causes relaxation of fibers, whereas LAT-B damages direct intercellular connections. In their study, Sureshkumar et al. [19] tested 48 human eye lens capsules that were collected 24 h postmortem. After extracapsular cataract extraction was performed, the bags were incubated in three types of solutions. The cells were then observed with photographic documentation for 28–30 days. In the control group, opacities formed on the lens capsule, leading to shrinkage of the capsule on days 3–5, which became more marked at the end of the observation period. No free or dead cells were observed in the surrounding fluid. In the group incubated with H-7, no PCO was detected during the observation period, whereas in the liquid medium, drifting free cells were visible (probably LECs that could not adhere to the capsule). Inhibition of growth of LECs on the capsule occurred at each drug concentration used. In the LAT-B group, the results varied according to concentration used. LECs were visible at a dose of 2 µM, some were visible, and some were not at a dose of 5 µM, and none were observed at a dose of 10 µM. There were no visible free cells floating in the medium in any subgroup. The differences in efficacy between the substances tested suggest that focal cell adhesion has a greater role than intercellular junctions in the formation of PCO. However, H-7 also inhibited wound healing in a rat cornea model [45], which would be an undesirable effect during postoperative recovery.

#### 1.7.3. EGFR as a Potential Target for Prevention of PCO

The epidermal growth factor receptor (EGFR) is of interest to researchers because it activates cascades that play a key role in the formation of PCO [46,47]. Stimulation of this receptor activates the tyrosine kinase cascade, which alters gene expression and increases cell activity and growth. Erlotinib and gefitinib are EGFR inhibitors that were registered and approved by the US Food and Drug Administration in 2003 and 2004, respectively, for the treatment of locally advanced or metastatic non-small cell lung cancer in combination with cisplatin and other cytostatic agents, and for advanced pancreatic cancer in 2007 [48]. 

The potential role of EGFR inhibitors in the prevention of PCO has been tested in vitro in human LECs (an HLE-B3 cell line) and spontaneously immortalized fetal cell line (an FHL-124 cell line) [45,46]. Their impact on the ability of these cells to proliferate, migrate, spread, and contract was investigated. At the same time, ex vivo observations were performed on human lens capsules. Cataract surgery was performed on the eyes of cadaveric donors 24 h postmortem. Pairs of collected anterior lens capsules were tested: one was incubated with a gefitinib/erlotinib solution and the other served as a control. Migration capacity was assessed as the ability of crystal violet-stained HLE-B3 cells to pass through a membrane with 8 µm pores and spread. PCO fundamentally involves proliferation of multiplied cells from the equator of the anterior lens capsule to the visual axis, which causes decrease in visual acuity. Both EGFR inhibitors decreased chemotactic migration, proliferation, and contractility of lens cells in vitro. In both cell culture and donor tissues, the migration of gefitinib-treated cells was prolonged from an average of 5.8 days to 10.6 days. Although no cytotoxicity was observed, these agents may cause trichomegaly, trichiasis, dysfunctional dry eye syndrome, and corneal ulceration with long-term oral administration.

#### 1.7.4. In Vitro Research Involving Photodynamic Therapy

Culturing human tissue is the model closest to in vivo conditions and can provide information on migration, proliferation, and differentiation of LECs [26].

Photodynamic therapy with bacteriochlorin has been studied as a potential method for prevention of PCO. Bacteriochlorin is obtained from the photosynthetic bacterium *Rhodospirillum rubrum* and acts as a photosensitizing agent [49]. Both photodynamic therapy and bacteriochlorin are safe when used separately but have a damaging effect on cells when combined in the presence of oxygen. This treatment strategy has been successfully used in the treatment of skin cancer, esophageal and bladder cancer, and non-cancerous diseases, such as arteriosclerosis and rheumatoid arthritis [50]. 

In a study by Fisher et al. [50], 106 human eyes were collected ex vivo and subjected to extracapsular cataract extraction. In the experimental group, bacteriochlorin was administered at concentrations of 50, 25, 12.5, 10, 6.25, 3.12, and 1.6 µg/mL for 10, 8, 6, 4, or 2 min with irradiation using laser diode light at a wavelength of 760 nm for periods of 12, 10, 5, or 2 min. Control groups were treated with bacteriochlorin only or light only. After 7 days of tissue culture, LECs underwent histological evaluation. In the experimental group that was incubated with 10 µg/mL bacteriochlorin for 10 min followed by irradiation for 15 min, proliferation activity and cell growth on the lens capsule was completely inhibited. After 7 days, no formation or growth of LECs was observed. The cell nuclei did not show staining or chromatin condensation. However, there were visible signs of apoptosis, and the cytoplasm showed signs of severe disorganization. These features were also observed, but to a lesser extent, in cells exposed to lower concentrations of bacteriochlorin and irradiation for a shorter period. In the control groups, LECs developed as a compact layer and contained heterochromatin-packed and euchromatin-packed nuclei and a well-differentiated cytoplasm with many cell organelles. The toxicity of bacteriochlorin to corneal endothelial cells and the ciliary body, as well as the duration of therapy, have yet to be established. Furthermore, the treatment period was extended by 25 min because this is the optimal application of both treatments. Bacteriochlorin could be administered as an intraoperative injection with application of light occurring after surgery.

#### 1.7.5. Ex Vivo Tests with Heat Shock Protein 90

Heat shock protein (HSP)90 is known to be involved in the regulation of cell proteostasis in the presence of pathological factors. However, its role in PCO is not known. Li et al. [51] investigated the potential therapeutic use of HSP90 in PCO using an LEC line and an ex vivo rat lens capsule. Protein expression in response to application of tanespimycin (17-AAG), an HSP90 inhibitor, was assessed by immunoblotting and real-time polymerase chain reaction (RT-PCR) and apoptosis by the TdT-mediated dUTP nick-end labeling method. Tanespimycin was found to suppress the proliferation of LECs and to inhibit the viability of the LECs remaining in the capsule. Moreover, tanespimycin induced apoptosis, which was characterized by an increase in reactive oxygen species levels, apoptotic DNA damage, and activation of caspase-3 and caspase-9. HSP90 has been reported to play a role in regulation of the EGFR and TGF-β receptor (TGFR) signaling pathways. Inhibition of HSP90 by tanespimycin led to the destabilization of EGFR and inhibition of TGF-β-mediated phosphorylation processes [45,46]. These data suggest that stimulation of HSP90 protects the cells remaining in the epithelium of the lens capsule from oxidative stress and is also involved in the regulation of cell proliferation, EMT, and migration of rat LECs through the EGFR and TGFR signaling pathways. Therefore, treatment with tanespimycin suppresses PCO and may be a candidate agent for prevention of PCO [45,46]. 

While in vitro studies are valuable, not all pathways of proliferation and migration, or contraction of the lens capsule are reproducible under laboratory conditions. Moreover, PCO in humans and laboratory animals may be stimulated to different degrees postoperatively depending on the operative course [52]. Therefore, in vivo tests are required.

## 2. Animal Models 

Rabbit eyes are suitable for assessment of the effectiveness of treatments that are potentially able to prevent PCO because opacities develop rapidly, usually within weeks after cataract surgery. Furthermore, the postoperative inflammatory reaction is rapid, and elevated levels of inflammatory markers persist for up to nine weeks after surgery [53,54]. 

### 2.1. Antimetabolites for Prevention of PCO

Antimetabolite agents have been widely studied for their ability to prevent PCO. They inhibit differentiation and proliferation of many cell types, including mitosis in LECs. Inan et al. [21] investigated the effect of retinoic acid and mitomycin C when administered intraoperatively to rabbit eyes during phacoemulsification. Of all retinoids, retinoic acid has the strongest inhibitory properties with respect to cell proliferation and collagen production by reducing production of TGF-β, and it is widely used to treat skin conditions and cancer. In ophthalmology, it has applications in xerophthalmia and experimental modulation of corneal wound healing. It has also demonstrated potential to inhibit cells in the retinal pigment epithelium during in vitro studies of PCO in laboratory animals [21,22]. Mitomycin C is an antineoplastic antibiotic derived from *Streptomyces caespitosus*. It is an alkylating agent, rather than an antimetabolite, and selectively inhibits DNA replication, mitosis, and protein synthesis. In the case of PCO, its action is mainly based on inhibition of fibroblast proliferation. In one study [22], rabbits were divided into three groups. In group I, retinoic acid was administered at a dose of 25 µg during hydrodissection and 225 µg into the lens capsule after the aspiration of the cortical masses. In group II, mitomycin C was administered at a dose of 0.04 mg; although this dose is lower than what is considered to be toxic (3.0 mg/mL), two subjects developed severe corneal edema, which led to their exclusion from further investigation. Group III served as the control group [22]. The PCO levels in groups I and II were significantly lower than those in the control group, with significantly fewer complications. 

The same authors extended their research to include three other substances [21]. More specifically, they investigated the effect of EDTA, arginine-glycine-aspartic acid (RGD) peptide, diclofenac, and dexamethasone in a rabbit eye model. EDTA is used mainly for diagnostic purposes. In the case of PCO, EDTA functions by chelating calcium ions, thereby inhibiting migration of LECs. It has been used to separate epithelial cells from the basal membrane in in vitro studies [55]. RGD, by contrast, inhibits integrins by competitively binding to the b-chain present in the ECM. Therefore, the LECs do not adhere to the lens capsule. After 3 months, the eyes underwent histological examination. All three substances had a significant inhibitory effect on formation of PCO in comparison with the control group. Mitomycin C was the most effective, followed by RGD, EDTA, and diclofenac. There was no statistically significant difference in the PCO scores between the test groups. The least effective was dexamethasone, although the PCO score in this group was significantly lower than that in the control group [20]. The most common postoperative complication was mild to moderate corneal edema, except for two eyes in the mitomycin C group, one eye in the EDTA group, and one eye in the diclofenac group; edema persisted for more than a week after surgery. Inflammatory cells were also observed in the aqueous humor of two eyes in the control group and one in the EDTA group. 

5-fluorouracil (5-FU) is a widely researched agent, and much hope has been raised for its use in the context of PCO inhibition. It is an antimetabolite that inhibits DNA synthesis and is active in the second phase of the cell cycle. It inhibits fibroblast proliferation, but adherence and migration remain unchanged.

Pandey et al. [56] designed a ring that slowly released 5-FU and implanted it into the lens capsule after the phacoemulsification procedure. It contained 300 g of 5-FU coated with poly(lactide-co-glycolide) (PLGA) to reduce the release of the drug. Seventeen New Zealand rabbits were divided into three groups (group 1, no ring implanted; group 2, the ring was implanted but without 5-FU; group 3, the ring containing the slow drug-release system was implanted). After eight weeks, statistically significant differences in the incidence of PCO were found between group 1 and the other two groups but not between groups 2 and 3. No damage was found in fragments of the retina or cornea that were collected to assess the cytotoxic effects of 5-FU on the surrounding tissues. However, the rabbit cornea exhibits much greater regenerative ability than the human cornea [57].

The effect of actinomycin on PCO was investigated by Nibourg et al., in a rabbit lens model [26] and by Sternberg et al., in an ex vivo human lens [58]. A combination of methotrexate and actinomycin D inhibited formation of PCO in both models. However, these findings were not confirmed by an in vivo study in monkeys [59]. 

Liu et al. [5] conducted a prospective randomized study of rapamycin with 60 New Zealand white rabbits. The animals were divided into three groups depending on the type of lens implanted: group A, a conventional lens; group B, a lens coated with a poly(lactic-co-glycolic acid) carrier only; and group C, a rapamycin-loaded PLGA-coated IOL. Histological observations showed that group C had the lowest accumulation of lenticular material on the posterior capsule, less inflammation in the anterior chamber after surgery, and less frequent and delayed PCO when compared with groups A and B. Rapamycin was detected in the anterior chamber through day 7 after surgery but not in the peripheral blood.

Nishi et al., conducted a similar study [60], which showed that the implantation of an indomethacin-soaked IOL significantly reduced formation of PCO in rabbit eyes, which had previously undergone phacoemulsification, during 12 months of follow-up. All indomethacin was released from the IOLs in BSS within 24 h. In another study, the same authors implanted a disc that gradually released indomethacin for 3 weeks after phacoemulsification surgery. Although inflammation of the anterior chamber was significantly reduced, indomethacin had no inhibitory effect on the formation of PCO [61].

Heparin is another agent that has been used in animal studies [62]. It is known to inhibit proliferation of LECs and fibroblasts, inhibit deposition and adhesion of platelets, macrophages, and fibroblasts on the surface of the posterior capsule, and reduce the activation of granulocytes, leading to reduced inflammation and activation of clot dissolution [2,9]. In one study, Xie et al., investigated the value of PLGA, a macromolecular carrier characterized by delayed release and good biodegradability, with 50 New Zealand white rabbits. The rabbits were divided into five groups: in group A, saline drops were administered; in group B, a DDS with the carrier was implanted into the posterior chamber of the eye; in group C, heparin eye drops were instilled in the eye; in group D, the DDS containing heparin was implanted in the subconjunctiva; and in group E, the DDS containing heparin was implanted into the posterior chamber of the eye. In all groups receiving heparin in any form, fewer and later signs of PCO were observed, while in group E, PCO did not occur in most cases (*n* = 6/10) during the entire 12-week observation period [63]. 

According to some researchers, only 5% of the volume of a drug administered via the conjunctiva can penetrate the eye due to the blood-aqueous humor barrier [4]. This limitation led to further research with the purpose of developing a DDS containing low-molecular-weight heparin for implantation in the posterior chamber as a safe and effective method of preventing PCO [64].

A postmortem study of 1% lidocaine without preservative in rabbit eyes conducted by Vargas et al. [65] showed that hydrodissection can reduce the number of viable LECs by reducing the adherence of cells to the basement membrane, damaging their desmosomal junctions, or by direct toxic effects. Sixteen eyes underwent phacoemulsification and were divided into two groups of eight: in group I, the anterior lens capsule was irrigated with 1% preservative-free lidocaine solution, and in group II the anterior lens capsule was irrigated with BSS for 1, 2, or 5 min. The capsules were stained with trypan blue and alizarin red. Photographic documentation was obtained and assessed for LEC damage. Fragments of the capsules from group I showed cytotoxic activity, which was manifested by blue-stained nuclei. The number of dead LECs increased with increasing duration of contact with lidocaine. This phenomenon was not observed in group II, regardless of irrigation time. Subsequently, in group II, four eyes underwent hydrodissection with 1% lidocaine and the other four with BSS followed by further standard phacoemulsification steps. The degree of LEC adhesion to the anterior capsule was assessed through histological examination, and in the group that underwent hydrodissection with lidocaine the anterior capsules were LEC-free. In the group treated with BSS, a normal layer of LECs was observed on the surface of the capsule. According to the subjective opinion of the surgeon, the process of cortical mass removal during irrigation/aspiration was easy in the group in which hydrodissection was performed with lidocaine. This effect can be explained by the weakening of cellular adhesion in the area of desmosomes, and thus decreased cell adherence. Furthermore, in vitro, lidocaine reduces production of leukotriene B4 and interleukin-1 and inhibits proliferation of fibroblasts, which may reduce formation of PCO. However, these promising results were not confirmed in human eyes, where it was shown that lidocaine reduced neither the incidence of PCO nor the inflammatory reaction in the anterior chamber in comparison with the control group [65].

### 2.2. Drug Delivery via Silicone Capsule

Maloof et al. created a sealed capsule irrigation device, the Perfect Capsule™ (Milvella Pty. Ltd., Epping, NSW, Australia). The device enables safe delivery and use of pharmacological agents [66] during cataract surgery while isolating the inner space of the capsule from the surrounding tissues. The system allows an experienced surgeon to safely rinse the intracapsular space after a sufficiently long application of a selected chemical preparation. 

Distilled water is a hypotonic solution that, through osmosis, can cause degeneration of hydrops, detachment of LECs from the basement membrane, nuclear disintegration, and cell lysis [67]. Crowston et al., suggested that the use of distilled water leads to rapid lysis of LECs in vitro without damaging the lens capsule, and can therefore be used in airtight irrigation systems during cataract surgery [67]. Using this technique in a rabbit model, they removed the lens by phacoemulsification and then irrigated the inner space with Triton X-100 (a nonionic surfactant that has a hydrophilic polyethylene oxide chain) and distilled water in group II without sealed-capsule irrigation (SCI) and in group III with SCI. Group I was the control group without irrigation [68]. After histological examination of the corneal endothelium, Descemet’s membrane, iris, and retina, they found that there were no statistically significant deviations from the normal condition within the groups or between groups I and II. However, in group II, they found extensive loss of corneal endothelial cells with remnants of scattered nuclei on Descemet’s membrane and lesions within the iris pigment layer. The lens capsule was wrinkled and contained both damaged and undamaged LECs with extensive cell lysis in the retina.

Fernandez et al., compared the effects of four substances previously tested for their ability to prevent PCO [69]. Twenty-eight rabbits were divided into two groups according to age (3–4 weeks, *n* = 16, and 6–9 weeks, *n* = 12). All eyes underwent phacoemulsification and 3 min of intraoperative intracapsular application of an ophthalmic viscosurgical device containing sodium hyaluronate (SHA) 1.4%, mitomycin C 0.2 mg/mL, EDTA 10 mM and 15 mM, 5-FU 33 mg/mL), acetic acid 3%, 0.3%, and 0.003%, BSS, or distilled water (for 1 min). 

To exclude the influence of the IOL on the formation of PCO, the eyes were left aphakic and the anterior capsule was closed with a miniature capsulorhexis valve manufactured specifically for the study. The observation period was five weeks (or less if advanced PCO was observed). All study groups developed PCO but at different rates. PCO occurred first in the SHA group after 1 day. PCO also occurred in the eyes treated with EDTA, mitomycin C, acetic acid 0.3% or 3%, distilled water (in younger animals), BSS, acetic acid 0.003%, 5-FU 10 mM, EDTA, and distilled water (in older animals) after 47 days. Other postoperative complications included corneal edema at the port site in the acetic acid group at both concentrations and appearance of inflammatory cells in the aqueous humor in the EDTA group. Eyes exposed to distilled water showed the most rapid regeneration and had the least severe inflammatory reaction after surgery. The results of this study did not confirm those of previous reports on the ability of EDTA to inhibit the formation of PCO [14]. In the EDTA group, appearance of PCO was somewhat delayed; however, by day 28, it was the same as that in the control group. The effectiveness of 5-FU was not as high as in other research that showed its effectiveness to be five times greater than that of a control group [70]. 

### 2.3. Controlled Cell Apoptosis

The problem of cataract and secondary clouding of the posterior capsule post-removal also occurs in dogs, reaching a frequency of 100% [71]. The pathomechanism is the same as that in humans. In a study by Pot et al. [71], selenium was applied to dogs’ lens capsules after euthanasia. Selenium is a catalyst for the formation of oxygen free radicals, which cause apoptosis. After reduction, the selenium anion enters a thiol oxidation reaction, the products of which are superoxide (O_2_^−^) radicals and hydrogen peroxide. Cells that die by apoptosis are removed by macrophages. The danger of widespread apoptosis is eliminated by the short decay time of the particles (<50 ms) and their small range of spread (<40 µm) [72]. The lens cells were cultured in vitro and divided into three groups. Group I cells were placed on a selenocystamine-coated IOL, group II cells were placed on an IOL without a coating, and group III cells were not placed on either type of IOL. The cells were observed and photographed for 10 days. In group I, the first symptoms of PCO appeared only on day six (on day one in the other groups) and were less severe. A cytotoxic effect was observed only at points where there was contact between the cells and the IOL.

### 2.4. Steroidal and Non-Steroidal Anti-Inflammatory Drugs

A study assessed the effect of steroidal and non-steroidal anti-inflammatory drugs on the formation of PCO in lenses explanted from rats [73]. The potential of these agents to prevent PCO derives from their ability to affect the production of TGF-β and bFGF, which have been shown to play a key role in the formation of PCO [28,74]. TGF-β and bFGF inhibit production of prostaglandins through different metabolic pathways. Non-steroidal anti-inflammatory drugs directly inhibit the release of the cyclooxygenase (COX) 1 and COX 2 isoforms. The action of steroids is more complex and involves the inhibition of phospholipase A2, a key enzyme in the synthesis of arachidonic acid, which is a precursor of prostaglandins and leukotrienes. Moreover, they participate in regulation of COX expression. 

In a study by Symonds et al., lenses from Wistar rats were cultured with the addition of TGF-β and bFGF to recreate the conditions under which PCO is formed [73]. The explanted lenses were then treated with dexamethasone and diclofenac. Both agents inhibited the formation of PCO by preventing the spread of abnormal cells along the lens capsule and accumulation of type I collagen in the ECM, with a progressive decrease in the number of abnormal cells. However, the smaller size of the rodent eye requires use of a different cataract removal technique. The inability to perform accurate continuous capsulorhexis or implant an artificial lens are serious limitations. There are also differences in the metabolic pathways involved [73,74].

Zukin et al., studied aldose reductase activity inhibition to attenuate induction of EMT markers in an in vivo model of cataract surgery [75]. Modified extracapsular lens extraction was performed in C57BL/6 mice and in mice with increased or decreased expression of aldose reductase. Immunofluorescence staining was used to assess the presence of markers of PCO postoperatively. Quantitative changes in expression of vimentin, fibronectin, E-cadherin, and α-SMA after surgery were evaluated by RT-PCR. Sorbinil, an aldose reductase inhibitor, was administered postoperatively. Changes in the number of EMT markers were assessed by RT-PCR. Inhibition of aldose reductase reduced the postoperative changes characteristic of EMT and protected against induction of lens fibrin cell markers.

## 3. Clinical Trials

Koopmans et al., demonstrated that the differences in metabolic pathways between species was the reason that pharmacological agents that have a positive effect in preventing PCO in animals are not necessarily effective in humans. Therefore, the effectiveness of an individual substance must be evaluated in clinical trials [59]. 

Rękas et al. [76] assessed the ability of distilled water to inhibit the formation of PCO. Using a procedure previously described by Maloof et al. [77], they examined 59 patients who underwent phacoemulsification, thorough polishing of the posterior capsule, and implantation of an IOL. Anticipating that it would not be possible to completely remove the LECs mechanically, additional irrigation of the inner surface of the capsule with distilled water suing the tight Perfect Capsule™ system was performed for 3 min in a second group of 29 eyes. A period of 3 min was used based on a preliminary finding that application of distilled water for 3 min causes cytolysis of cells with a probability of 70.8%, (i.e., 3.51 times greater than would be achieved in 1 min). The purpose of using distilled water was to remove and/or significantly damage as many LECs as possible on the inner surface of the lens capsule to limit their proliferation and migration, thereby preventing secondary cataract formation. Irrigation allowed clean, transparent pouches to be obtained from the lens without the residues of lens masses and LECs visible under the operating microscope.

In this study, the use of a liquid formulation made it possible to safely reach every part of the capsule. Application of distilled water (a hypo-osmotic fluid) damaged the LECs by osmosis, leading to complete cytolysis or significant cell damage [68]. The few residual LECs were damaged and had low mitotic, proliferative, and migratory capacity, which reduced the risk of developing PCO. Twenty-four months after the procedure, PCO was less advanced in the eyes treated with distilled water and occurred in a smaller area of the capsule than in the eyes where only mechanical cleaning of the capsule was performed. The safety profile in both groups was the same. There were no statistically significant between-group differences in the rate of complications such as corneal edema, Descemet’s membrane folds, corneal epithelial erosion, or postoperative astigmatism. Endothelial cell loss was slightly higher in the distilled water group; however, this finding could not be attributed to the use of distilled water alone and was more likely due to mechanical manipulation during insertion and removal of the Perfect Capsule™ system. Unlike in the study by Crowston et al. [67], use of distilled water in vitro did not produce signs of damage to the lens capsule on histological examination. Therefore, it seems that this substance can be used in SCI systems during cataract surgery. The disadvantage of its use in SCI is the extended operating time, additional manipulations in the anterior chamber during implantation and explantation of the Perfect Capsule™ system, as well as the cost of purchasing the system [2]. 

D’Antin et al. compared the effect of hydrogen peroxide and distilled water on the progression of PCO [78]. Human lens capsules were divided into three groups—one treated with hydrogen peroxide, another with distilled water, and a control group. The lens capsules in the active treatment groups were rinsed for 5 min in 30 mM hydrogen peroxide solution or distilled water. Samples were then grown for a month, during which time microscopic images were obtained. Specimens were subjected to overall histological evaluation with hematoxylin-eosin staining and immunohistochemical evaluation of α-SMA, Ki-67, and vimentin. The authors observed growth retardation in the groups treated with hydrogen peroxide and distilled water in comparison with the control group, with no significant differences in growth between the two groups.

Joshi and Hussain studied the effect of irrigation of the lens capsule with trypan blue (0.06%) on PCO in 100 eyes that had undergone phacoemulsification from 50 patients with senile cataracts [79]. One eye in each patient was randomized to one of two groups. The study group received a 0.2-mL injection of trypan blue into the lens capsule after cleaning of the cortical layer. The control group (the second eye of the same patient) received a 0.2-mL injection of BSS administered in a similar manner to the trypan blue. PCO was assessed in the central zone of the implanted lens using customized computer software at 6, 12, 24, and 36 months postoperatively. At 6 months, there was a reduction in the degree of PCO in the trypan blue group. However, no treatment effect was observed at 36 months. 

Joshi et al., assessed the effect of rotation of the implanted lens/IOL by 360° during cataract phacoemulsification [80]. After placing/inserting the lens/IOL in the capsule, the lens was rotated 90° using a Sinskey hook inserted through the corneal incision. The anterior chamber was filled with viscoelastic. Another Sinskey hook was then inserted through the side port, and the lens was rotated 180°. A third hook was introduced through the port on the opposite side, and the IOL was rotated 90°. The effect was analyzed after 6, 12, 24 and 36 months in the group with IOL rotation and compared with a control group in which no rotation was performed. Postoperative follow-up was performed by slit-lamp examination in mydriasis. Median PCO scores at 6, 12, and 24 months were significantly lower in the rotation group than in the control group. There was no significant difference between the two groups during observation months 24–36. The authors concluded that rotation of the IOL in the capsule is a simple and safe method for preventing PCO and requires no additional tools or operator skills.

## 4. Local Administration of Steroid and Non-Steroidal Anti-Inflammatory Drugs after Surgery

Use of anti-inflammatory drugs for prevention of PCO is also under investigation. These agents inhibit the production of prostaglandin E2, which plays a role in the formation of PCO.

In vitro studies have confirmed that diclofenac suppresses proliferation and metaplasia of LEC fibers [81] and inhibits the synthesis of prostaglandin E2 and collagen [82]. However, the data from in vivo studies are inconclusive. 

Seki et al. showed that administration of diclofenac three times daily for a year inhibited the formation of PCO [83]. In contrast, a prospective, randomized, double-blind study by Neumayer et al., that investigated the effect of a topical combination of diclofenac and prednisolone on the formation of Elschnig’s pearls found no significant difference between the active and placebo groups [84]. Moreover, other long-term studies of diclofenac and dexamethasone did not confirm a benefit during 2–3 years of observation [85,86,87]. Similar results were obtained in a study comparing diclofenac with betamethasone [88]; however, the duration of administration was shorter (a maximum of 3 months) in that study. Another retrospective study in 120 patients compared the occurrence of PCO 3 years after treatment with diclofenac 0.1% or ketorolac tromethamine 0.5% and found no significant between-group differences in frequency of PCO (12% in both groups). However, when compared to the occurrence rate in the general population, this is a favorable result [89].

Tranilast (N-[3’,4’-dimethoxycinnamoyl]-anthranilic acid) inhibits the collagen synthesis that occurs because of the proliferation of fibroblasts from hypertrophic scars or keloids without affecting the healing processes in normal, healthy skin. This oral antiallergic agent reduces vascular permeability and inhibits the release of chemokines, such as TNF-β, from mast cells and other inflammatory cells. Tranilast drops are widely used in Japan for allergic conjunctivitis and have few known side effects [90]. 

Miyazawa et al. hypothesized that the inflammatory process would favor the penetration of tranilast into damaged tissues and found high concentrations of the drug in the aqueous humor up to 30 min after intraocular administration in rabbits that had undergone phacoemulsification four weeks earlier [91]. 

A prospective, randomized, double-blind study by Tobari et al. [90] evaluated the ability of tranilast drops to prevent PCO in humans when administered into the conjunctival sac. Forty-four eyes that had undergone cataract phacoemulsification were randomized postoperatively and administered either tranilast 0.5% or placebo (vehicle only) four times a day for three months. Standard postoperative ofloxacin and dexamethasone therapy was also provided for 1 week after surgery. Patients were examined and photographic documentation was obtained one week, one month, and three months after the procedure. The intensity of the PCO was determined by the degree of light scattering at 10 points in the posterior capsule, and the result was calculated by the algorithm in digital form. After three months of observation, the PCO level was significantly higher in the placebo group. However, the trial design had no effect on corneal wound healing. Table 1 summarizes the characteristics of the most impactful studies discussed in our review.

## 5. Discussion

Efforts to prevent PCO are ongoing. However, the pharmacological agents used intraoperatively should be effective and safe. The outstanding issue is the toxicity of substances used to treat LECs and their effect on other structures and tissues of the eye, in particular the corneal endothelial cells. Several pharmacological agents have been found to damage LECs in both animals and humans. The type and extent of damage varies according to type of pharmacological preparation and duration of exposure. The more cytotoxic the agent, the more rapid and greater the damage to LECs. Therefore, despite the promising results of in vitro studies, the use of chemicals (namely 5-FU and mitomycin C) that damage LECs by inhibiting their proliferation is limited. Non-steroidal anti-inflammatory drugs are safer because they have the potential to reduce PCO, especially when used in sustained-release systems, at lower concentrations, and with a a longer duration of action. Their main mechanism of action is toxic damage to LECs. Furthermore, in vitro experiments using substances that inhibit TGF-β have yielded promising results. However, at this stage, disruption of metabolic pathways is certain to affect other tissues as well, and the in vivo consequences are difficult to predict. Compounds affecting LECs by means of osmotic pressure, such as 23.4% sodium chloride and distilled water, offer greater possibilities for use in clinical practice. The use of a technique that enables easy, rapid, and safe delivery to the inside of the lens capsule and removal of toxic substances, allowing for selective damage to LECs, may be the best way to overcome the present difficulties in preventing PCO. 

## 6. Conclusions

Despite many efforts, an effective, economical, and optimal pharmacological method for the prevention of PCO that is accessible and could be widely used in patients is yet to be developed. The application of hydrogels, which have the desired physical properties (namely, porosity), may be an option for use in DDSs for sustained release of the loaded drug while reducing its toxic side effects on endothelial cells. Further research is needed to elucidate the exact pathogenesis of PCO, to establish the most appropriate human and animal cell lines for use as part of in vitro models, and to create animal models that will help in the search for an effective and safe pharmacological strategy for PCO.

## Figures and Tables

**Table 1:** Clinical research for PCO.

Substance Used	Cell Culture	Tissue Culture	Animal Experiments	Clinical Trials	Reference	Methodology
Lidocaine 1%			+		Vargas et al., 2003 [65]	16 rabbit eyes subjected to phacoemulsification postmortem
EGFR inhibitors: Erlotinib Gefitinib	+				Wertheimer et al., 2013, 2014, 2015 [46,47,48]	HLE-B3 and FHL-124 cell cultures and lens pouches collected from human donor eyes ex vivo
Bacteriochlorin		+			Van Tenten et al. [49]	106 human eyes: bags underwent PDT with BCA as the photosensitizer
Dexamethasone Diclofenac		+			Symonds et al. [73]	Lenses explanted from rat eyes and incubated with TGF-β and FGF
5-Fluorouracil	+		+		Pandey et al. [56] Nibourg et al. [26] Sternberg et al. [58]	Rabbit lenses, human lens ex vivo
Mitomycin C			+		Inan et al. [21]	Administered intraoperatively to rabbits during phacoemulsification
Methotrexate			+		Sternberg et al. [58]	In vivo in monkeys
Celecoxib and Rofecoxib		+			Chandler et al. [92] Davis et al. [93]	Lenses removed from dogs ex vivo
Rapamycin	+	+			Liu et al. [5]	Lens implanted in white rabbits
Cyclosporin A	+		+		Cortina et al. [82] Totan et al. [94] Pei et al. [95]	Human LEC passage Cataract surgery in rabbits Cataract surgery in rabbits with a sustained delivery system
Heparin			+	+	Rönbeck et al. [96] Wejde et al. [97] Maedel et al. [98] Xie et al. [63]	Prospective study comparing incidence of PCO following implantation of various IOLs in humans Study in rabbit eyes implanted with a heparin-coated IOL
Antibodies TGF-β SNAIL siRNA CAT-152 Pirfenidone	+	+			Li et al. [36] Yang et al. [38]	Human LECs Lens capsules

BCA, bacteriochlorin; EGFR, epidermal growth factor receptor; FGF, fibroblast growth factor; IOL, intraocular lens; LEC, lens epithelial cells; PCO, posterior capsule opacification; PDT, photodynamic therapy; TGF-β, transforming growth factor-beta; siRNA, small interfering RNA.

## Data Availability

All materials and information are available upon e-mail request from the corresponding author.
